# Influenza D Virus: Serological Evidence in the Italian Population from 2005 to 2017

**DOI:** 10.3390/v12010030

**Published:** 2019-12-27

**Authors:** Claudia M. Trombetta, Serena Marchi, Ilaria Manini, Otfried Kistner, Feng Li, Pietro Piu, Alessandro Manenti, Fabrizio Biuso, Chithra Sreenivasan, Julian Druce, Emanuele Montomoli

**Affiliations:** 1 Department of Molecular and Developmental Medicine, University of Siena, Via Aldo Moro, 53100 Siena, Italy; serena.marchi2@unisi.it (S.M.); ilaria.manini@unisi.it (I.M.); emanuele.montomoli@unisi.it (E.M.); 2 VisMederi srl, Strada del Petriccio e Belriguardo 35, 53100 Siena, Italy; kistner@vismederi.com (O.K.); piu@vismederi.com (P.P.); 3 Department of Biology and Microbiology, South Dakota State University, Brookings, SD 57007, USA; feng.li@sdstate.edu (F.L.); Chithra.Sreenivasan@sdstate.edu (C.S.); 4 VisMederi Research srl, Strada del Petriccio e Belriguardo 35, 53100 Siena, Italy; alessandro.manenti@vismederiresearch.com (A.M.); biuslee@yahoo.it (F.B.); 5 Victorian Infectious Diseases Reference Laboratory, 792 Elizabeth Street, Melbourne, VIC 3000, Australia; julian.druce@mh.org.au

**Keywords:** influenza D virus, seroprevalence, humans, Italy

## Abstract

Influenza D virus is a novel influenza virus, which was first isolated from an ailing swine in 2011 and later detected in cattle, suggesting that these animals may be a primary natural reservoir. To date, few studies have been performed on human samples and there is no conclusive evidence on the ability of the virus to infect humans. The aim of this serological study was to assess the prevalence of antibodies against influenza D virus in human serum samples collected in Italy from 2005 to 2017. Serum samples were analysed by haemagglutination inhibition and virus neutralization assays. The results showed that the prevalence of antibodies against the virus increased in the human population in Italy from 2005 to 2017, with a trend characterized by a sharp increase in some years, followed by a decline in subsequent years. The virus showed the ability to infect and elicit an immune response in humans. However, prevalence peaks in humans appear to follow epidemics in animals and not to persist in the human population.

## 1. Introduction

Influenza D virus (IDV), a novel influenza virus, was first isolated from an ailing swine in 2011 in Oklahoma, USA [1,2]. Although the viral genome shows approximately 50% overall homology with influenza C virus (ICV), no cross-reactivity with antibodies directed against human ICV and no re-assortment with human ICVs have been observed so far. Moreover, attempts to detect viable recombinant progeny involving ICV and IDV have not been successful [1,3,4,5].

Several epidemiological and serological studies have reported the isolation of IDV in cattle from many geographic areas (Canada, the United Kingdom, Japan, the United States, Mexico, Luxemburg, Ireland, France and China), suggesting that cattle may be a primary natural reservoir for the virus [6,7,8,9,10,11,12,13,14,15,16,17,18]. In addition, two studies have suggested that IDV has circulated among beef cattle since at least 2003/2004 [9,13]. IDV has also been identified in other animal species, such as sheep, goats, camelids and horses across countries in different continents (Europe, North America, Africa and Asia), but there is no evidence of infections in chickens and turkeys [18,19,20,21,22,23,24].

The prevalence of antibodies against IDV in domestic pigs ranges from 9.5% to 11.7% [1,25], indicating that the virus is able to circulate among domestic pigs, but is not yet widespread [26]. A similar IDV antibody prevalence has also been reported in horses in the Midwest United States [22]. A higher seroprevalence (19.1%) has been found in feral pigs, which could have been due to the increased chances of having contact with various domestic and wild animals and, hence, a higher exposure to IDV [27]. Seroprevalence of antibodies against IDV increases substantially especially in newborn calves, as a result of maternal antibodies, and in older cattle. As the level of maternal antibodies declines, calves become more susceptible to IDV infection; this increases the risk of active transmission and the potential to create a virus reservoir [9,12,13,26]. Moreover, IDV can be efficiently transmitted among cattle by direct contact [10]. The presence of IDV in pigs and cattle has also been reported in Italy, where it has been confirmed by PCR and virus isolation, as well as serological analysis for the presence of IDV specific haemagglutination inhibition (HI) antibodies [25,28]. Specifically, a study conducted in the swine population in Northern Italy revealed a high prevalence (11.7%) of antibodies against IDV in 2015, which demonstrated significantly increased seroprevalence compared to the reported rate in 2009 (prevalence 0.6%) [25].

To date, few studies have been performed on human samples. A screening study of human serum samples showed that 1.3% of the general population had antibody titres against IDV [1], while in Scotland no evidence of IDV infection emerged from the analysis of archived respiratory samples [29]. However, a serological study conducted in Florida reported a seroprevalence of 94% among workers exposed to cattle (32/35 samples) [30].

Although there is no conclusive evidence that IDV can infect humans, a study conducted in ferrets, which are the preferred human surrogate animal models for influenza virus studies, has shown that the virus is able to spread among ferrets and that it has a broader cellular tropism than human ICVs [1]. These features indicate that IDV carries the risk of becoming a potential threat to public health.

The aim of this serological study was to assess the prevalence of antibodies against IDV in archived human serum samples collected in Italy from 2005 to 2017.

## 2. Materials and Methods

### 2.1. Influenza Viruses

Influenza D/bovine/Oklahoma/660/2013 virus was originally isolated from the bovine herds of Oklahoma.

Influenza C/Victoria/2/2012 virus was originally isolated in 2012 from a nasopharyngeal swab of a child with clinical symptoms of acute respiratory infection and was submitted for virus isolation to the Victorian Infectious Diseases Reference Laboratory, Melbourne, Australia. The virus was isolated after cultivation in Madin Darby Canine Kidney (MDCK) cells at 33 °C, together with RT-PCR diagnosis and sequence confirmation.

IDV and ICV were propagated in MDCK cells, using UltraMDCK serum-free medium (SFM) supplemented with 2 µg/mL of acetylated trypsin (IDV) and trypsin (ICV) from bovine pancreas (Sigma-Aldrich, Saint Louis, MO, USA) and 100 IU/mL penicillin-streptomycin.

Cells were seeded in a T175 cm^2^ culture flask at a density of 1 × 10^6^ cells/mL with UltraMDCK SFM. After 18–20 h, the cell monolayer was washed twice with sterile Dulbecco’s phosphate buffered saline (DPBS). After the DPBS had been carefully removed, cells were infected with 3.5 mL (IDV) or 10mL (ICV) of UltraMDCK SFM (without Trypsin) containing the respective virus at a multiplicity of infection of 0.001. After 1 h of incubation at 33 °C in a humidified atmosphere with 5% CO_2_, 50 mL of UltraMDCK SFM containing a final concentration of 2 µg of trypsin acetylated (IDV) and 0.5 µg of TPCK (ICV) was added to the flask. The infected cells were incubated at 33 °C in a humidified atmosphere with 5% CO_2_ for 36 h. At the end of the incubation period, additional acetylated trypsin was added up to a final concentration of 1 µg/mL and the flask was incubated at 37 °C in a humidified atmosphere with 5% CO_2_ for another 36–48 h. The cytopathic effect (CPE) was monitored every day, along with the hemagglutination (HA) titre of the supernatant. At 90% of the CPE, the culture medium was harvested, centrifuged at 4 °C in order to remove the cell debris, and stored at −80 °C.

### 2.2. Serum Samples

Archived human serum samples from adults (≥18 years old) were obtained from the Serum Bank of the Laboratory of Molecular Epidemiology, Department of Molecular and Developmental Medicine, University of Siena, Siena, Italy.

The samples were anonymously collected in Tuscany (Central Italy) and Apulia (Southern Italy) and stored in compliance with Italian ethics law. The only information available was the age of each subject and the year of sampling. A total of 1281 serum samples collected from 2005 to 2017 (approximately 100 serum samples for each year) were randomly selected, though a balanced distribution between males and females and among age-groups in each year was ensured, according to the availability of serum samples for each year (Table S1).

Influenza D (swine) and C (rooster) hyperimmune serum samples, kindly provided by Istituto Zooprofilattico Sperimentale della Lombardia e dell’ Emilia Romagna (IZSLER, Brescia, Italy) and Institut National de la Recherche Agronomique (INRA) (France) and Ecole Nationale Vétérinaire de Toulouse (INP-ENVT) (France), were used as positive controls.

Human serum without IgA, IgM and IgG was used as a negative control (Sigma-Aldrich, S5393).

### 2.3. Haemagglutination Inhibition Assay

The HI assay was performed as described in Hause et al. [1]. All serum samples, including positive and negative controls, were pre-treated with receptor-destroying enzyme from Vibrio Cholerae (Sigma Aldrich, Milano, Italy) (ratio 1:5) followed by heat inactivation for 1 h at 56 °C. Serum samples were tested in duplicate by using turkey red blood cells (0.35%). The antibody titre was expressed as the reciprocal of the highest serum dilution that showed complete inhibition of agglutination. Since the starting dilution was 1:10, when the titre was below the detectable threshold, the results were conventionally expressed as 5 for calculation purposes [31].

### 2.4. Virus Neutralization Assay

The MDCK cell cultures were grown at 37 °C in 5% CO_2_ and pre-incubated in a 96-well plate for 4 h.

Serum samples, including positive and negative controls, previously heat-inactivated at 56 °C for 30 min and tested in duplicate, were two-fold diluted with EMEM culture medium supplemented with 0.5% fetal bovine serum in a 96-well plate and mixed with an equal volume of virus (100 TCID50/well). After 1 h incubation at 37 °C in 5% CO_2_, the mixture was added to the MDCK cell suspension (1.5 × 10^5^ cells/mL). Plates were read for HA activity in the supernatant after three days of incubation at 37 °C in 5% CO_2_.

### 2.5. Statistical Analysis

According to the definition of seropositivity used for other newly emerging viruses, all positive serological responses (HI titres ≥1:10) were classified as seropositive, while non-detectable HI responses (<1:10) were regarded as negative and arbitrarily expressed as a value of 5 [31]. In addition, positive titres were classified in positive (≥1:10, ≥1:20), and highly positive (≥1:40). For the purpose of direct comparison of HI and virus neutralization (VN) assays, the proportions for each category of titres were calculated by applying the total number of serum samples tested in the HI and VN assays. Calculation of the 95% confidence intervals for the proportions was based on the Clopper–Pearson exact method [32].

For positive and highly positive titres, the Pearson’s Chi-squared test was used to verify the significant differences among the proportions of titres between the two assays across years, and to make yearly-based comparisons of the observed proportions for each assay. The Marascuilo procedure was used for the post-hoc analysis, which accounts for multiple comparisons. Relative changes of proportions were evaluated in relation to the value of the proportion measured for 2005, the first year tested, which was used as the base year for the analysis. The Hodrick–Prescott filter, with a smoothness penalty parameter *λ* = 1600, was implemented over the time series of the relative changes in proportions in order to estimate their trend components [33]. All the analyses were made as two-sided tests and conducted at a significance level of 5%. RStudio (version 1.1.463) was used for all the statistical analyses.

## 3. Results

### 3.1. Investigations on Potential Cross-Reactivity between IDV- and ICV-Positive Serum Samples

The specificity of the HI assay with respect to the potential cross-reactivity between IDV and ICV was evaluated by testing both viral antigens against IDV and ICV specific hyper-immune antisera generated in swine and rooster. As shown in Table 1, no cross-reaction between IDV and ICV was observed. The anti-serum specific for the D/bovine/Oklahoma/660/2013 strain showed a high HI titre (1:10,240) as did the anti-serum specific for the C/Victoria/2/2012 (HI titre 1:640). No HI titres were detectable when the hyper-immune antisera were tested against the respective heterologous influenza C or D strain. These results demonstrated the specificity of the IDV HI assay and were therefore used for the analysis of the presence of IDV-specific antibodies in human serum samples.

The IDV hyperimmune serum sample was used as positive control in HI and VN assays showing a titre range of 2560–10,240 and 1280–5120, respectively.

### 3.2. Analysis of Human Serum Samples for the Presence of IDV-Specific Antibodies

A total of 1281 human serum samples, collected randomly from adults in the Italian regions of Tuscany and Apulia from 2005 to 2017, were tested by HI assay in order to detect the presence of antibodies against IDV. The results clearly show that IDV specific HI antibodies were present in at least a small subset of serum samples taken in every single year between 2005 and 2017, although IDV was isolated and described for the first time in 2011 ((A) in Table 2). IDV antibodies displayed low levels, between 5.1% and 9.8%, in the years 2005–2007, followed by a sharp increase in 2008; the highest levels (33.9–46.0%) were reached in 2008, 2009, 2010, 2013, 2014 and 2016, while the lowest levels (11.9–25.7%) were seen in 2011, 2012, 2015 and 2017.

In addition, the highest levels of HI seropositivity (HI titres ≥ 1:40) were found in serum samples collected in 2008, 2009, 2012, 2013, 2014 and 2016. The human sera with positive HI titres (≥1:10) were then tested in an IDV specific VN assay to confirm the positive HI titres and subsequently the specificity of the HI assay for IDV ((B) in Table 2).

### 3.3. Pearson’s Chi-Squared Test for Multi-Proportions

Differences between the HI and VN assays proportions in homologous class of titres were not significant. By contrast, the analysis of proportions among years showed significant differences for both assays in each class of titres (Table S2). Comparisons of the titre proportions over years were conducted on each class of titres except the negative class, as this class is overtly complementary to the positive (≥1:10) class of titres (Table S3). In the HI assay, the proportions of titres ≥1:10 in the first three years (2005–2007) proved to be almost always lower than the values measured in the other years. The proportion observed in the year 2017 significantly differed only when compared with the values recorded for the years 2010–2014.

Likewise, in the VN assay, the proportions in the years 2005 and 2006 proved to be almost always different from the proportions in the other years. The 2007 value, however, was significantly different only when compared with the values for the years 2013–2014, as well as the 2017 value. Significant differences in the proportions of HI titres ≥1:20 were identified only between the peak values (in 2008, 2009, 2013, 2014) and the values recorded in the first two years, 2005 and 2006. Regarding the class of highly positive titres (≥1:40), there was not enough evidence to identify significance on pairwise comparisons of proportions.

**Figure 1:**
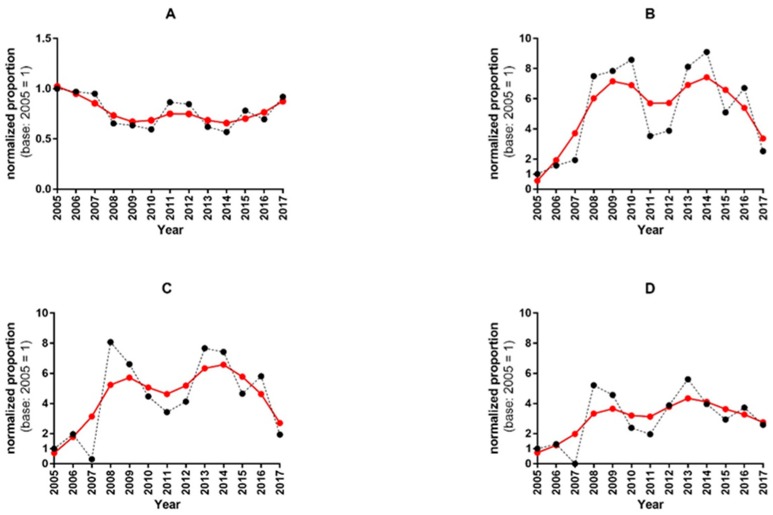
Time course of the normalized proportions and their trend lines in HI titres. The panels (**A**–**D**) display the normalized proportions (black dots) of negative (5), positive (≥1:10, ≥1:20), and high positive (≥1:40) HI titres respectively, with the trend curves (red dots) estimated by using the Hodrick–Prescott filter. Panel (**A**): the dynamics of the time series shows values that are almost always lower than the base 2005 values. After 2014, the trend seems to have turned upwards towards the initial base level. Panel (**B**): the time series of the positive titres (≥10) shows a bimodal pattern, with peaks in 2010 and 2014, preceded by huge upsurges in 2008 and 2013. Peaks in the trend curve were observed in 2009 and 2014. Panel (**C**): the positive titres ≥20 show a similar bimodal pattern in the time series, with peaks in normalized proportions occurring in 2008 and 2013. The trend curve shows two peaks in the same years as that of the positive titres ≥10. Panel (**D**): the time series of the normalized proportions of highly positive titres (≥1:40) displays its highest values in 2008 and 2013. In the trend curve, the first peak is delayed by one year (2009), and the second peak coincides with the year 2013.

Our analysis of the relative changes in proportions provided insights into the dynamics of the proportions of titres. Figure 1 shows the normalized proportions of negative, positive and highly positive HI titres and their corresponding trend curves.

Over the years, the proportion of the negative HI titres was always lower than the base value of 2005 (94.9%). The positive titres showed similar bimodal patterns, with relative minima in 2011, 2015 and 2017. The proportion of the positive titres (≥1:10) peaked in 2010 and 2014. The proportions of positive titres ≥1:20 and ≥1:40 showed an absolute minimum in 2007 and an upsurge in 2008 and 2013, a trend that differed slightly from that of the ≥1:10 positive titres.

The curves of the normalized proportions of the titres, as well as their trend curves, displayed similar characteristics in the HI and VN assays over the period 2005–2017 (Figure S1).

## 4. Discussion

The detection and isolation of IDV in pigs with influenza-like clinical signs and respiratory distress in 2011, and the evidence of a seroprevalence of 1.3% of humans [1] in combination with the circulation of IDV in pigs in Northern Italy which was confirmed in 2015 [25], have raised the question of whether IDV antibodies can be found in the Italian population since this potential outbreak. This hypothesis is supported by a study conducted in Florida by White et al., which reported a seroprevalence of 94% among workers exposed to cattle [30].

The results of the present study indicate that the findings of studies conducted in various countries also apply to Italy. The low seroprevalence (5.1%) of IDV specific antibodies found in the human serum samples from the first year of the study (2005) suggests that IDV may have circulated at least in Italy already before 2005. This finding is supported by a study by Luo et al. [13], which suggested that Nebraska beef cattle had been exposed to IDV since at least 2003, and that the virus may have already circulated at least 8 years before its detection.

The potential IDV circulation in pigs in Northern Italy in 2015 [25], which was confirmed by PCR analysis, positive virus isolation, and increased IDV specific HI antibody responses in animals from the farms affected, may have been associated with an increase in IDV HI-specific antibodies in humans from 25.7% in 2015 to 33.9% in 2016. These data also indicate that there could have been undetected IDV circulation in pigs and/or cattle already in 2007 and reflect a confirmed outbreak in cattle in 2011 [7], since the seroprevalence of HI specific antibodies in humans increased from 9.8% in 2007 to 37.9% in 2008 and from 19.6% in 2012 to 41.0% in 2013.

These data clearly show that seroprevalence against IDV increased in Italy from 2005 to 2017. This increase was not constant over the years, it shows sharp rises in some years followed by drops in subsequent years. The seroprevalence peaks detected in humans appear to follow IDV epidemics in animals, as an epidemic in cattle in France in 2011 [7] and an outbreak in pigs in Italy in 2015 [25] have been reported. Moreover, as the titres did not remain high in the years following the increase, but dropped to lower levels, we could speculate that a spill over event from an animal reservoir occurred, and that the virus does not circulate primarily in humans. However, IDV proved able to elicit an immune response in humans. The background seropositivity rate in the population was higher in 2017 (11.9%) than in 2005 (5.1%), although this difference did not prove statistically significant. Follow-up studies over the next couples of years should be performed in order to determine whether the titres drop further to levels below 10% over the years or remain at a higher level than that observed in 2005. In addition, cattle and pigs should be carefully observed and analysed with respect to potential new outbreaks, which could be followed by an increase in IDV-specific antibodies in humans in the subsequent year.

Although IDV was first isolated from a diseased pig in 2011 [1], this virus is notable for being the first influenza virus identified in cattle [3]. This is supported by evidences of past infection in cattle from the same farm as the diseased pig. Finally, additional studies revealed cows to be the primary reservoir [3,9,10,11,12]. Since then, IDV has been isolated from cattle and pigs in several countries, including China, the United States and France [3,4,5,6,7,8], and antibodies against the virus have been found in sheep, goats, horses and camelids in China, Ethiopia, France, Japan, Mongolia, Ireland and the United States [19,20,21,22,23,24].

The origin and ecology of IDV remain unknown. Although it is not yet known how or when IDV first emerged, analysis of archived serum samples by SJCEIRS (St. Jude Children’s Research Hospital Center of Excellence for Influenza Research and Surveillance) suggests that IDV has been circulating in cattle since at least 2004 [2,9,10,13]. IDV infections in cattle also tend to be associated with other respiratory infections, particularly pneumonia, but the significance of this observation is not known [8].

To date, no indications that IDV can cause disease in humans have been found, but several groups have already signalled its potential threat as an emerging pathogen in specific target groups, such as cattle-workers [5], or as a considerable public health risk [2,5,13,26,34,35]. This eventuality is supported by a number of findings by different groups. One of the main risks stems from the ability of the virus to infect and to be transmitted to a number of domestic mammal species, such as cattle, pigs, goats, sheep and camelids, and also to wild animals, such as feral pigs [22]. In addition, it has been shown that IDV can infect ferrets, the gold standard for influenza studies in animals [1,26], and guinea pigs, as shown by transmission studies [26,36].

Seroprevalence studies in humans have shown that the risk of transmission from infected cattle to humans may be very high. Indeed, seroprevalence rates of 91% (HI assay) and 97% (VN assay) have been documented in cattle-workers, as opposed to 18% (on VN assay) in control subjects without contact with cattle [26,30]. The results of the present study show seroprevalence peaks in humans that seem to follow IDV epidemics in animals (outbreak in pigs in Italy in 2015 [25]). However, further studies on the circulation of IDV in animals in the same years would be useful to support this hypothesis. The increased risk of transmission to humans is supported by the finding that the IDV hemagglutinin-esterase-fusion glycoprotein exhibits an open receptor-binding cavity, which forms the basis for its broad cell tropism and, consequently, its broad host tropism [37]. A valuable tool for studies on IDV replication kinetics and cell tropism has been provided by Holwerda et al. [38], who used “primary well-differentiated human airway epithelial cells” as an in vitro respiratory epithelium model of humans.

At least three antigenic lineages of IDV have been identified—D/Oklahoma, D/660 and D/Japan—and HI analysis have shown up to a 10-fold loss in cross-reactivity against heterologous antiserum [8,39,40]. Serological analysis in this study has been conducted using D/660-like strain; however, the use of a single IDV strain may ultimately lead to an underestimation of the true seroprevalence in Italian population, leaving the conclusions of this study unchanged.

Despite the solid results yielded by a considerable number of studies, our current knowledge of IDV is still limited, and neither potential threats to exposed individuals nor public health issues can be fully excluded. Consequently, additional research on IDV and diligent observation of IDV prevalence in the various animal hosts and in potentially affected individuals should be conducted in the future.

## Figures and Tables

**Table 1:** HI cross-reactivity between IDV and ICV against hyper immune sera.

Influenza Viruses	IDV Antiserum HI Titre	ICV Antiserum HI Titre
D/bovine/Oklahoma/660/2013	1:10,240	5
C/Victoria/2/2012	5	1:640

**Table 2:** IDV specific HI (A) and VN (B) titres of human serum samples collected from 2005 to 2017 in Italy.

	**HI Assay**														
**Year**		**2005**	**2006**	**2007**	**2008**	**2009**	**2010**	**2011**	**2012**	**2013**	**2014**	**2015**	**2016**	**2017**	**Total**
**Samples**		99	101	82	95	101	83	101	102	100	100	101	115	101	1281
**Titre**	5	94	93	74	59	61	47	83	82	59	54	75	76	89	**946**
	≥1:10	5	8	8	36	40	36	18	20	41	46	26	39	12	**335**
	≥1:20	4	8	1	31	27	15	14	17	31	30	19	27	12	**236**
	≥1:40	3	4	0	15	14	6	6	12	17	12	9	13	8	**119**
	≥1:80	2	1	0	7	9	3	2	8	7	2	2	4	5	**52**
	≥1:160	1	0	0	5	2	0	2	0	2	0	0	3	3	**18**
N≥1:10		5	8	8	36	40	36	18	20	41	46	26	39	12	**335**
%≥1:10		5.1%	7.9%	9.8%	37.9%	39.6%	43.4%	17.8%	19.6%	41.0%	46.0%	25.7%	33.9%	11.9%	**26.2%**
CI 95% lower		1.7%	3.5%	4.3%	28.1%	30.0%	32.5%	10.9%	12.4%	31.3%	36.0%	17.6%	25.3%	6.3%	**23.8%**
CI 95% upper		11.4%	15.0%	18.3%	48.4%	49.8%	54.7%	26.7%	28.6%	51.3%	56.3%	35.4%	43.3%	19.8%	**28.6%**
(**A**)															
	**VN Assay**														
**Year**		**2005**	**2006**	**2007**	**2008**	**2009**	**2010**	**2011**	**2012**	**2013**	**2014**	**2015**	**2016**	**2017**	**Total**
**Samples**		5	8	8	36	40	36	18	20	41	46	26	39	12	335
**Titre**	5	0	1	0	2	7	16	1	5	2	4	4	9	4	**55**
	≥1:10	5	7	8	34	33	20	17	15	39	42	22	30	8	**280**
	≥1:20	5	5	8	33	18	17	15	13	29	35	35	20	7	**240**
	≥1:40	4	4	2	10	2	5	9	9	12	14	14	7	4	**96**
	≥1:80	1	1	0	1	0	1	2	0	4	0	0	0	1	**11**
	≥1:160	0	0	0	1	0	0	2	0	1	0	0	0	0	**4**
N≥1:10		5	7	8	34	33	20	17	15	39	42	22	30	8	**280**
%≥1:10		5.1%	6.9%	9.8%	35.8%	32.7%	24.1%	16.8%	14.7%	39.0%	42.0%	21.8%	26.1%	7.9%	**21.9%**
CI 95% lower		1.7%	2.8%	4.3%	26.2%	23.7%	15.4%	10.1%	8.5%	29.4%	32.2%	14.2%	18.3%	3.5%	**19.6%**
CI 95% upper		11.4%	13.8%	18.3%	46.3%	42.7%	34.7%	25.6%	23.1%	49.3%	52.3%	31.1%	35.1%	15.0%	**24.2%**
(**B**)															

## Supplementary Materials

The following are available online at https://www.mdpi.com/1999-4915/12/1/30/s1, Figure S1: VN vs. HI, normalized proportions and trend lines for the titre class ≥1:10, Table S1: Serum samples collected in Italy from 2005 to 2017, Table S2: Results of the multiple proportion test among years, Table S3: Multiple comparisons of proportions of positive titres (≥1:10, ≥1:20) for HI and VN assays along the period 2005–2017.
