# Supplementary material for: Influenza D Virus: Serological Evidence in the Italian Population from 2005 to 2017
Source: Viruses. 2019 Dec 27;12(1):30. doi: 10.3390/v12010030 (PMC7019439; doi:10.3390/v12010030)
Supplement: Supplementary file 1 [file viruses-12-00030-s001.pdf]

Supplementary file

Table S1. Serum samples collected in Italy from 2005 to 2017.

**Year**		**2005**	**2006**	**2007**	**2008**	**2009**	**2010**	**2011**	**2012**	**2013**	**2014**	**2015**	**2016**	**2017**
Samples		99	101	82	95	101	83	101	102	100	100	101	115	101
**Age groups**	18-30	10	13	7	10	16	7	14	16	7	9	15	10	10
	31-40	19	14	14	18	17	14	18	19	16	15	16	16	17
	41-50	14	14	14	14	15	14	13	11	19	24	15	27	25
	51-60	18	15	15	16	18	16	16	18	18	16	12	20	19
	61-70	18	13	14	17	13	15	14	12	20	13	18	18	12
	71-80	14	14	11	14	14	11	16	14	14	10	17	14	10
	≥81	6	18	7	6	8	6	10	12	6	13	8	10	8
Sex	M	50	52	41	48	55	43	49	52	50	50	51	50	51
	F	49	49	41	47	46	40	52	50	50	50	50	65	50

Table S2 Results of the multiple proportion test among years.

Assay	Titre class	Chi-squared	df	p value
HI	negative	131.22	12	< 0.0001
	≥1:10	131.22	12	< 0.0001
	≥1:20	86.31	12	< 0.0001
	≥1:40	34.95	12	0.0005
VN	≥1:10	105.78	12	< 0.0001
	≥1:20	86.32	12	< 0.0001
	≥1:40	23.32	12	0.02

Table S3. Multiple comparisons of proportions of positive titres (≥ 1:10, ≥ 1:20) for HI and VN assays along the period 2005-2017.

**HI positive titres (≥ 1:10)**			**HI positive titres (≥ 1:20)**			**VN positive titres (≥ 1:10)**			**VN positive titres (≥ 1:20)**		
value	*CR*	*contrast*	*value*	*CR*	*contrast*	*value*	*CR*	*contrast*	*value*	*CR*	*contrast*
0.33	0.25	2005-2008	0.29	0.24	2005-2008	0.31	0.25	2005-2008	0.31	0.24	2005-2008
0.35	0.24	2005-2009	0.23	0.22	2005-2009	0.28	0.24	2005-2009	0.25	0.23	2005-2013
0.38	0.27	2005-2010	0.27	0.23	2005-2013	0.34	0.25	2005-2013	0.31	0.24	2005-2014
0.36	0.25	2005-2013	0.26	0.23	2005-2014	0.36	0.25	2005-2014	0.28	0.25	2006-2008
0.41	0.25	2005-2014	0.31	0.23	2007-2008	0.22	0.21	2005-2016	0.28	0.25	2006-2014
0.29	0.23	2005-2016	0.26	0.21	2007-2009	0.29	0.25	2006-2008	0.28	0.25	2008-2017
0.30	0.26	2006-2008	0.30	0.22	2007-2013	0.26	0.24	2006-2009	0.28	0.25	2014-2017
0.32	0.25	2006-2009	0.29	0.22	2007-2014	0.32	0.25	2006-2013			
0.35	0.28	2006-2010	0.22	0.19	2007-2016	0.34	0.25	2006-2014			
0.33	0.26	2006-2013				0.29	0.27	2007-2013			
0.38	0.26	2006-2014				0.31	0.27	2007-2014			
0.26	0.24	2006-2016				0.28	0.26	2013-2017			
0.28	0.27	2007-2008				0.30	0.27	2014-2017			
0.30	0.27	2007-2009									
0.34	0.29	2007-2010									
0.31	0.27	2007-2013									
0.36	0.27	2007-2014									
0.31	0.29	2010-2017									
0.28	0.27	2013-2017									
0.33	0.27	2014-2017									

Table shows differences in proportions, the corresponding critical value from the Marascuilo procedure (CR), and years compared. A difference greater than the CR indicates a statistically significant result. Only the significant differences for each class of positive titres in both assays are reported.

Figure S1. VN vs. HI: normalized proportions and trend lines for the titre class ≥1:10.

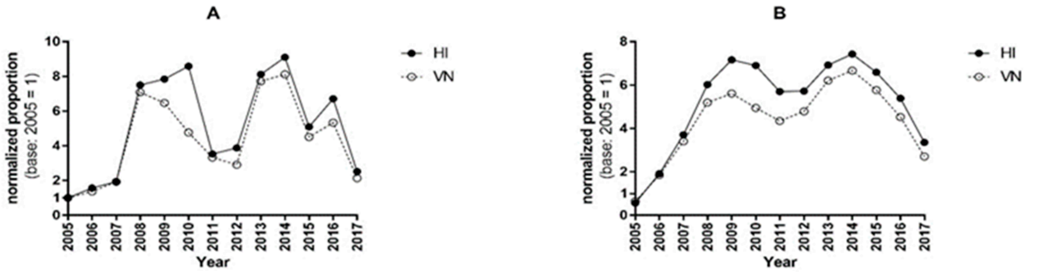

Panel A: Time series of the normalized proportions of positive (≥1:10) titres of VN assay (empty black dots) compared to the time series of the HI assay (black filled dots).
Panel B: Trend curves related to the normalized proportions of positive (≥1:10) titres for the VN assay (empty black dots) and the HI assay (black filled dots).
